# Phylogenetic Analysis of Cellulolytic Enzyme Genes from Representative Lineages of Termites and a Related Cockroach

**DOI:** 10.1371/journal.pone.0008636

**Published:** 2010-01-08

**Authors:** Nemuri Todaka, Tetsushi Inoue, Kanako Saita, Moriya Ohkuma, Christine A. Nalepa, Michael Lenz, Toshiaki Kudo, Shigeharu Moriya

**Affiliations:** 1 Laboratory of Environmental Molecular Biology, Graduate School of Yokohama City University, Yokohama, Kanagawa, Japan; 2 Bio Resource Center, RIKEN Tsukuba Institute, Wako, Saitama, Japan; 3 Department of Entomology, North Carolina State University, Raleigh, North Carolina, United States of America; 4 Termite Research, CSIRO Entomology, Canberra, Australia; 5 Biosphere Oriented Biology Research Unit, RIKEN Advanced Science Institute, Yokohama, Kanagawa, Japan; Georgia Institute of Technology, United States of America

## Abstract

The relationship between xylophagous termites and the protists resident in their hindguts is a textbook example of symbiosis. The essential steps of lignocellulose degradation handled by these protists allow the host termites to thrive on a wood diet. There has never been a comprehensive analysis of lignocellulose degradation by protists, however, as it has proven difficult to establish these symbionts in pure culture. The trends in lignocellulose degradation during the evolution of the host lineage are also largely unknown. To clarify these points without any cultivation technique, we performed meta-expressed sequence tag (EST) analysis of cDNA libraries originating from symbiotic protistan communities in four termite species and a wood-feeding cockroach. Our results reveal the establishment of a degradation system with multiple enzymes at the ancestral stage of termite-protistan symbiosis, especially GHF5 and 7. According to our phylogenetic analyses, the enzymes comprising the protistan lignocellulose degradation system are coded not only by genes innate to the protists, but also genes acquired by the protists via lateral transfer from bacteria. This gives us a fresh perspective from which to understand the evolutionary dynamics of symbiosis.

## Introduction

Cellulosic biomass is now regarded as a very exciting candidate source for bio-fuel. Currently, the use of ethanol as fuel incurs a cost to the food supply, as ethanol production requires both starch and sucrose. Woody biomass can be used as an alternative, but doing so entails the daunting challenge of saccharifying cellulose with the enzymes from the biomass. The most crucial step is the treatment of lignin, a component that resists enzymatic degradation and prevents enzymes from accessing cellulose. The symbiotic relationship with the protistan community within the termite gut seems to endow termites with the ability to degrade cellulose from complex natural ligno-cellulose that is composed of lignin, hemi-cellulose and cellulose [1]–[3]. The termite system may thus provide useful clues for the establishment of an artificial process for saccharifying woody biomass.

Termites and the protists that reside in their hindguts are a classic example of symbiosis, and the essential steps of lignocellulose degradation that is handled by symbiotic protists allow the host termites to thrive on a wood diet. Because of this relationship, the termite has a highly efficient cellulose degradation system. This system draws energy from woody biomass by degrading cellulose without degrading lignin. The termite selectively incorporates cellulose, and 80% to 90% of the cellulose incorporated is exchanged to acetate. Interestingly, termites possess their own cellulase family but these proteins are insufficient to provide for the energy needs of the termite. If the symbiotic system is lost, a termite can not survive under cellulose feeding conditions, meaning that termites are completely dependent upon their symbionts for biomass degradation. [1]–[5]

As of this writing, our understanding of the biochemical process underlying this saccharification system derives solely from the estimations of a few biochemical studies and PCR-based analyses [6]–[15]. Among the various glycosyl hydrolase families (GHFs) responsible for lignocellulose degradation, only three families have been identified from the symbiotic systems of termite, in a limited number of termite species. Enzymes of families 5 and 7 were found in the symbiotic system of *Coptotermes formosanu*s, and an enzyme of family 45 was found in the symbiotic systems of *Reticulitermes speratus* and *Mastotermes darwiniensis*, although these host termites were also extensively investigated as the source of the cellulases (reviewed in reference [1]). These findings are too incomplete to draw up a complete schema of the efficient ligno-cellulose degradation system of the termite.

Cellulose is generally degraded by several different families of cellulases. These cellulases efficiently degrade ligno-cellulose through synergistic mechanisms. A synergistic reaction of enzymes is very likely to take place in the protistan consortium. This symbiotic relationship is not between one organism and another, but between multiple symbionts. And to complicate matters further, almost all of these protists are difficult to cultivate. The complexity and inaccessibility of the cellulolytic system of the symbiotic protistan consortium make it difficult to obtain and understand whole components of this effective cellulolytic system [2], [3]. This is why only a few cellulase families were found from limited species of protists and host termites. If we can comprehensively obtain the cellulase genes from a whole symbiotic protistan community in the termite gut and reconstruct the evolutional trail of the cellulolytic system along the termite lineage, we may be able to identify the actual core components of termite systems for the efficient degradation of cellulose.

To circumvent this difficulty, in this current report, we did not rely on an ordinary one-by-one cloning strategy to elucidate the complete system of the multi-organism consortium. Instead, we constructed a cDNA library from a mixed protistan population and performed a comprehensive set of expressed sequence tag (EST) analyses of cDNA libraries. Recently, we published a similar analysis about the symbiotic system of *Reticulitermes speratus* [16]. Here, we attempt to understand the evolutionary history of the cellulolytic system of the termite lineage by using phylogenetic analyses in addition to the previously established meta-transcriptome strategy to investigate the termite symbiotic system. For this purpose, cDNA libraries were constructed from four termite species representative of the overall lineage of lower termites, and from *Cryptocercus punctulatus*, a wood-feeding cockroach thought to be a sister taxon to the termite lineage.

Our results showed that all of the symbiotic systems investigated here share the two families of cellulase genes, GHF5 and 7, and some more enzyme genes, GHF10, 11 and 45, may help this “core enzyme set” in several species of investigated symbiotic systems. Interestingly, our result indicates that a core gene, GHF5, was laterally transferred from bacteria to the protistan symbiotic system of the termite lineage at the most ancient stage of their evolution.

## Results and Discussion

We investigated the trends in lignocellulose degradation during the evolution of the host lineage using four representative termite species from four families of wood-feeding termites (*Reticulitermes speratus* (Rhinotermitidae), *Hodotermopsis sjostedti* (Termopsidae*), Neotermes koshunensis* (Kalotermitidae), and *Mastotermes darwiniensis* (Mastotermitidae)) and *Cryptocercus punctulatus*, a wood-feeding cockroach that is a member of a genus thought to be in a sister clade to the termite lineage [17].

In total, we obtained 910 clones (*R. speratus*), 920 clones (*H. sjostedti*), 1056 clones (*N. koshunensis*), 1021 clones (*M. darwiniensis*), and 868 clones (*C. punctulatus*) as ESTs from the cDNA libraries constructed from the symbiotic protistan communities of the host insects. The ESTs of the data sets were annotated by homology searches with fastx analysis [18] using the DDBJ web site (http://fasta.ddbj.nig.ac.jp/) and clustered into 752, 839, 716, 711, and 762 possibly orthologous clusters. All of the sequences obtained were screened for potential contaminants. Instead of detecting the bacterial structural genes by homology analysis, a difficult challenge in light of the high diversity of the sequences, we tried to detect the ribosomal RNA sequences in the library using blast analysis [19]. Our screening by this method revealed the following contaminant ratios of ribosomal RNA: 0.40%, 4.89%, 0.42%, 0.84%, and 0.79%, respectively. We concluded, based on these results, that our cDNA library was almost completely constructed by the poly T and CAP selection method. The foregoing homology analyses revealed that homologues of glycosyl hydrolases responsible for lignocellulose degradation were predominantly expressed in each host-symbiont system (Table 1). The glycosyl hydrolases have been classified into more than 80 families based on amino acid sequence similarities [20], [21]. Among these, homologues of GHFs 5, 7, 10, 11, and 45 are found within these symbiotic systems. According to blast analysis, the e-values of these homologues ranged from 1.00e–117 to 2.00e–9 against the top known cellulase gene hit for each.

**Table 1:** The number of GHF clones from EST analysis.

	Cellulase				Xylanase						
Host	GHF5	GHF7EG	GHF7CBH	GHF45	GHF8	GHF10	GHF11	GHF43	GHF62	# GHF's	Total
*R. speratus*	8	21	41	4	1	0	6	3	1	85	910
*H. sjostedti*	19	19	43	8	0	10	7	0	0	106	920
*N .koshunensis*	16	54	20	0	1	72	0	0	1	164	1056
*M. dawinensis*	5	15	20	44	1	23	0	0	0	113	1021
*C. punctulatus*	12	1	17	1	0	14	0	0	0	98	868

Next, we conducted a preliminary phylogenetic analysis using one-pass sequences homologous to GHF5, 7, 10, 11, and 45 based on the EST analysis and selected representative clones of each GHF. We then determined 154 full-length sequences of these representatives for a further phylogenetic analysis (GHF5 = 45 clones, GHF7 = 39 clones, GHF10 = 38 clones, GHF11 = 8 clones, GHF45 = 24 clones). All of the 154 GHF sequences contained a poly A sequence on the 3′ end, as well as the initial and terminal codons. In the case of cDNAs from bacteria, which may also have a poly A tail, we would not expect to see start and terminal codons as the poly A tail is a signal that targets the transcript for degradation by endonucleases. Therefore, any cDNAs in our samples transcribed from bacterial mRNA that possessed a poly A sequences can be expected to not contain the complete functional domain sequence and terminal codon [22]. This, coupled with the low overall contamination rate based on 16S ribosomal RNA (above) confirms that all of the GHFs obtained were retrieved from symbiotic protists but not bacteria.

In relation to the characteristics of the GHF homologues, the residues corresponding to the putative catalytic nucleophile (Glu) and general acid/base (Glu) were completely conserved among the homologues of GHF5 [23]. Likewise, the putative catalytic nucleophile and general acid/base among the homologues of GHF7, 11, and 45 were completely conserved [24]–[26]. In the analysis of GHF10, the catalytic nucleophile (Glu) and general acid/base (Glu) were conserved in 35 homologues but unassignable in 3 [27]. However, the sequences of these three unassignable homologues and *Clostridium acetobutylicum* xylanase GHF10 (UniProt accession number Q97TP5) showed 71.2–71.6% amino acid similarity.

**Figure 1:**
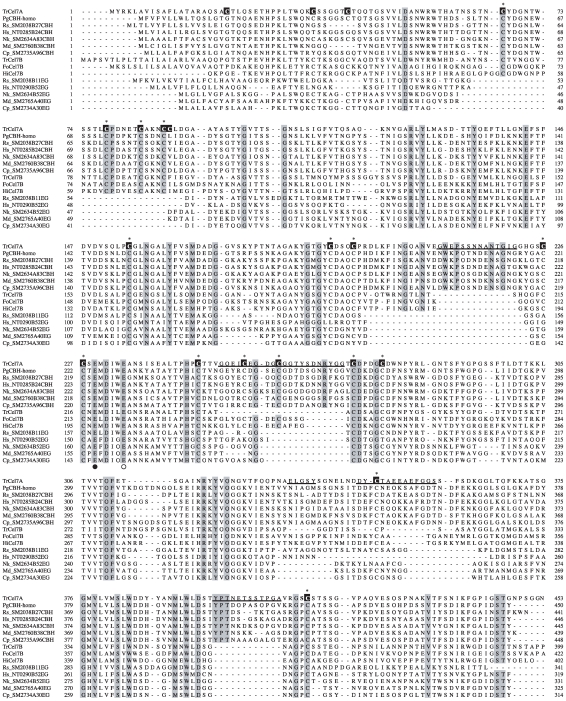
Multiple alignments of symbiotic protist cellulase and catalytic domains of the members of the glycoside hydrolase family 7. Rs, *Reticulitermes speratus symbiotic protists*; Hs, *Hodotermopsis sjostedti symbiotic protists*; Nk, *Neotermes koshunensis symbiotic protists*; Md, *Mastotermes darwiniensis symbiotic protists*; Cp, *Cryptocercus punctulatus symbiotic protists*; Numbered clones (for example SM2038A27) are clone names of cellulase genes identified in this study from the symbiotic protists of termites; CBH, cellobiohydrolase; EG, endoglucanase; TrCel7A, a cellobiohydrolase component, *Trichoderma reesei* Cel7A [Uni Prot. P00725]; PgCBH-homo, *Pseudotrichonympha grassii* PgCBH-homo1 [Q95YH1]; TrCel7B, an endo-β-1,4-glucanase (EG) component, *T. reesei* Cel7B [P07981]; FoCel7B, an EG component, *Fusarium oxysporum* Cel7B [P46237]; HiCel7B, an EG component, *Humicola insolens* Cel7B [P56680]. The alignments were performed using CLUSTAL_W and subsequent manual refinement based on the three-dimensional structures of reference sequences. Arabic numerals denote the number of residues from each N terminal end. Solid and open circles under the column indicate the sites of putative proton donors and general acids/bases, respectively. Shaded columns represent conserved positions within the sequences. White letters with black shading denote cysteine residues composing the disulfide bond of *T. reesei* Cel7A. The asterisks represent the putative protistan GHF7 CBH homologue cysteine residue sites corresponding to the cysteine residue sites of *T. reesei* Cel7A. The underlined sequences in TrCel7A indicate the loop-forming regions covering the catalytic tunnel [22], [23].

Seventy-seven full-length sequences homologous to GHF7 cellulase were identified. The clone Rs38B27CBH (DDBJ accession number AB274538) homologous to GHF7, for example, included the putative catalytic nucleophile (Glu221) and general acid/base (Glu226) (Fig. 1). These homologues of GHF7 could be divided into two groups, namely, those with insertion sequences and those without them. The GHF7 cellulases take the form of either cellobiohydrolases (CBHs) or endoglucanases (EGs) The CBHs are known to include insertion sequences that make up a tertiary cellulose binding tunnel structure [28], [29]. As shown in Fig. 1, the regions corresponding to the insertion sequences *in T. reesei* Cel7A were conserved in homologues of GHF7 CBH. Their sequences also shared the 16 cysteines forming the disulfide bonds found in *T. reesei* Cel7A. Disulfide bonds are essential for stabilizing the GHF7 CBH tertiary structure. These results strongly suggest that the genes obtained from the cDNA libraries encode the active enzymes of each GHF.

The homologues of GHF5, 7, and 45 were previously found to be of a symbiotic protist origin [6]–[8], [11], [12]. These earlier demonstrations did not, however, include any inferences on the evolutionary roots of lignocellulose degradation in termites. In contrast, this current study clearly indicates that a degradation system with multiple enzymes was established at the ancestral stage of termite-protistan symbioses. According to our results, GHF 5 and 7 were obtained from all symbiotic systems investigated. GHF10 was absent in *Reticulitermes*, while GHF11 was obtained only from the subterranean termites *Reticulitermes* and *Hodotermopsis*. GHF45 was absent only in *Neotermes*.

This result clearly indicates that GHF5 and 7 predate or evolved concomitant with extant termite symbiotic systems and that these genes existed during the whole evolutionary history of these systems. It also implies that these two GHFs are core enzymes of the highly efficient cellulolytic system in termites. In the case of GHFs 10, 11 and 45, if we hypothesize that our EST analysis has enough coverage, GHF10 was secondarily lost at or just after the node that branches *Reticulitermes* from the main termite lineage and GHF45 was secondarily lost in *Neotermes* after branching from the main termite lineage. Also, GHF11 was obtained from a subterranean termite (Table 1). To clarify this attractive hypothesis, we are now undertaking a high throughput EST sequencing project using massive pyrosequencing technology.

Phylogenetic analyses of the homologues of the GHFs were performed by the maximum likelihood method with wide taxon sampling. We used a rooted topology for phylogeny reconstruction of GHF5 and 7 because the sub-family structures were known. In the case of the other phylogenies, we have no information that allows us to root the trees. Therefore we used star shape topologies for those phylogenetic reconstructions.

**Figure 2:**
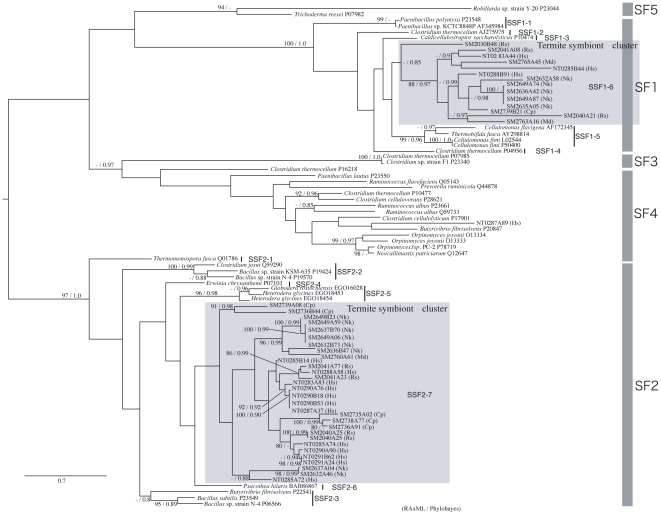
Phylogenetic tree of GHF5. Numbered clones (for example SM2030B48) are cellulase genes identified in this study from the symbiotic protists of termites. Letters in parentheses after each clone denote host termite species (Rs  =  *Reticulitermes speratus*, Nk  =  *Neotermes koshunensis*, Hs  =  *Hodotermopsis sjostedti*, Md  =  *Mastotermes darwiniensis*, Cp  =  *Cryptocercus punctulatus*). Accession numbers of reference sequences are denoted after species names. SF are indicated the sub-family (21) and SSFs are indicated sub-sub families that were used in Fig. 3. In sub-family 1 and 2, gray boxed sub-sub-family indicated that composed by symbiont's sequences and other sub-sub-families composed by bacterial sequences.

**Figure 3:**
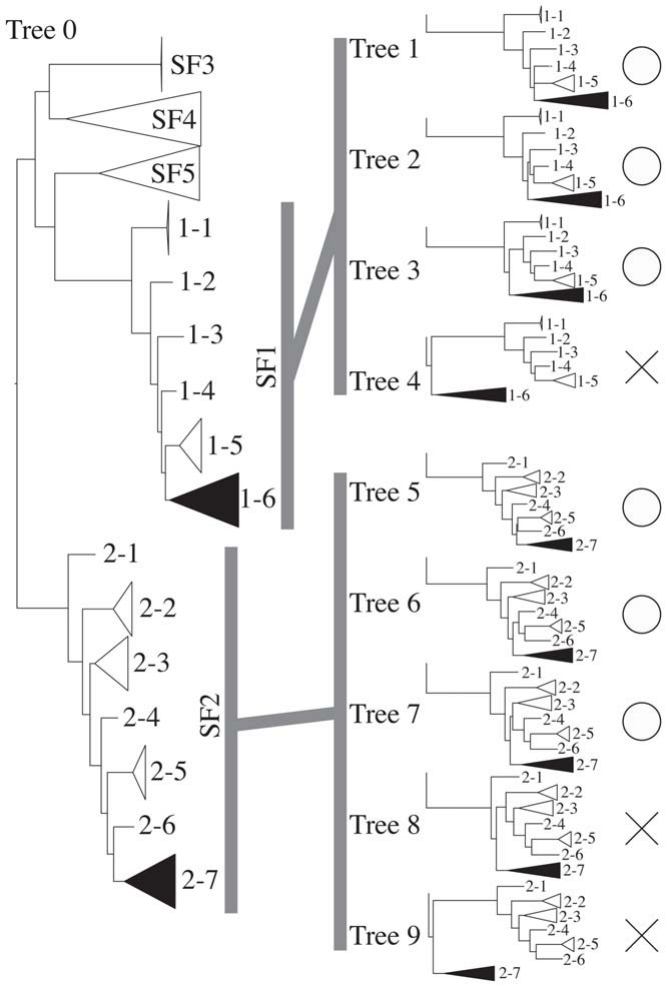
Tree topologies for the SH test of GHF5. Tree 0 is the original topology that was inferred by RAxML. From 1–1 to 2–7 were indicated the sub-sub-families that were showed in Fig. 2 (SSF1–1 - SSF2–7). SSF1–6 and 2–7 were composed with the symbiont sequences of sub-family 1 and 2, respectively. Cross marks and open circles indicate that the topology can be rejected or not, respectively.

Based on our results GHF5 can be divided into 5 sub-families, as shown by a previous study [23]. Our phylogenetic tree only showed a difference in that while sub-families 3 and 4 are also here each inferred as independent monophyletic clusters, this inference is without high supporting values. The sequences obtained here from the symbiotic systems of termites and cockroaches were mainly assigned to sub-families 1 and 2. Interestingly, as shown highlighted by gray boxes in Fig. 2, both these symbiont clusters (“sub-sub-families (SSF)”) located inside the nodes basal to sub-families 1 and 2. Both of these sub-families are composed of bacterial sequences and these results indicate that our symbiont sequences formed a cluster nested within these bacterial clusters. These nested clusters were not supported by ML bootstraps or Bayesian posterior probabilities. Therefore, we performed the Shimodaira-Hasegawa (SH) test for these nested topologies (Fig. 3 and Table 2). The results showed that both of the nested topologies inside the bacterial cluster can not be rejected statistically (Table 2). This topology and unexpected nested/sister taxonomic relationship between the eukaryotic symbiont genes and prokaryotic genes suggests that lateral gene transfers from bacteria to symbiotic protists have occurred. These termite symbiont clusters contained homologues from *Cryptocercus* and all four of the host termite species. On this basis, we speculate that two gene transfers may have occurred during the early stages of the evolution of the symbioses.

**Table 2:** Results of the SH test of phylogenetic trees for GHF5 endoglucanase.

Tree	Likelihood	D (LH)	SD	Significantly Worse
Original tree	−23656.138725	0.000002	0.003925	No
Sub-family 1 (SF1)				
Tree: 1	−23661.508283	−5.369557	5.259180	No
Tree: 2	−23664.186747	−8.048020	6.056825	No
Tree: 3	−23669.184360	−13.045633	7.034359	No
Tree: 4	−23672.529571	−16.390844	7.795750	Yes
Sub-family 2 (SF2)				
Tree: 5	−23656.224424	−0.085698	5.196696	No
Tree: 6	−23664.193831	−8.055104	8.352466	No
Tree: 7	−23685.341415	−29.202688	13.402450	Yes
Tree: 8	−23693.223915	−37.085188	15.092283	Yes
Tree: 9	−23694.477411	−38.338684	15.109157	Yes

We also identified an isolated homologue from *Hodotermopsis* (NT0287A89) in sub-family 4 within the bacterial cluster (Fig. 2). In this cluster, several rumen fungal sequences also co-located. Although high resolution was not obtained, there is the possibility that sub-family 4 may also have been laterally transferred from bacteria to termite symbionts and rumen fungi. Further extensive research will clarify this interesting phenomenon.

**Figure 4:**
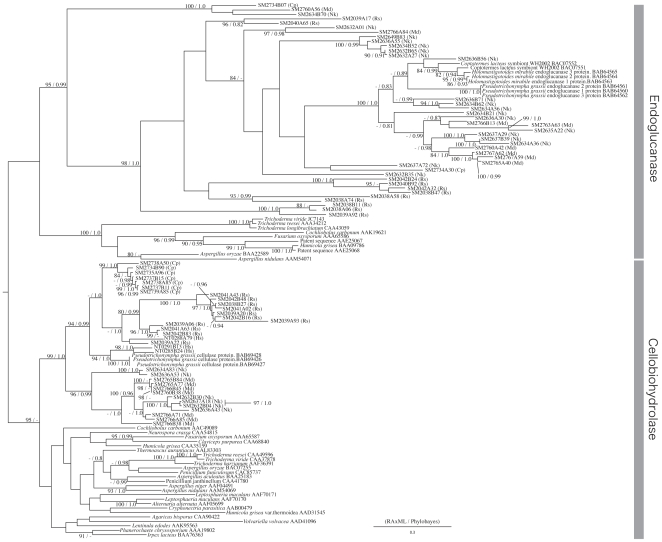
Phylogenetic tree of GHF7. Numbered clones (for example SM2734B07) are cellulase genes identified in this study from the symbiotic protists of termites. Letters in parentheses after each clone denote host termite species (Rs  =  *Reticulitermes speratus*, Nk  =  *Neotermes koshunensis*, Hs  =  *Hodotermopsis sjostedti*, Md  =  *Mastotermes darwiniensis*, Cp  =  *Cryptocercus punctulatus*). Accession numbers of reference sequences are denoted after species names.

On the other hand, as shown in Fig. 4, the members of GHF7 could be divided into two large clades, one corresponding to the EGs and the other to the CBHs. According to our result, these two main sub-families were well supported by high bootstrap values. Interestingly, the GHF7 homologues from our cDNA libraries formed monophyletic clusters within each clade with high supporting values (Fig. 4).

The symbiont clusters in both the CBH and EG clades consisted of homologues from the cDNA libraries of all of the host termite species and *Cryptocercus*. Those clusters also include several published sequences from termite symbionts that are hosted by *Coptotermes formosanus*, and grouped separately from fungal sequences in both cases. Thus, this result suggests that the GHF7 homologues of symbiotic protists in lower termite can be defined as an independent sub-clade of enzymes within the GHF7 cellulase sub-family.

The topology of the GHF7 tree is completely different from the GHF5 tree. The result of the GHF7 phylogeny showed that eukaryotic sequences and prokaryotic sequences clearly divided into independent clusters in each of the subfamilies of GHF7. This suggests that the GHF7 gene does not originate from lateral gene transfer from bacteria but that it evolved along with organismal evolution.

**Figure 5:**
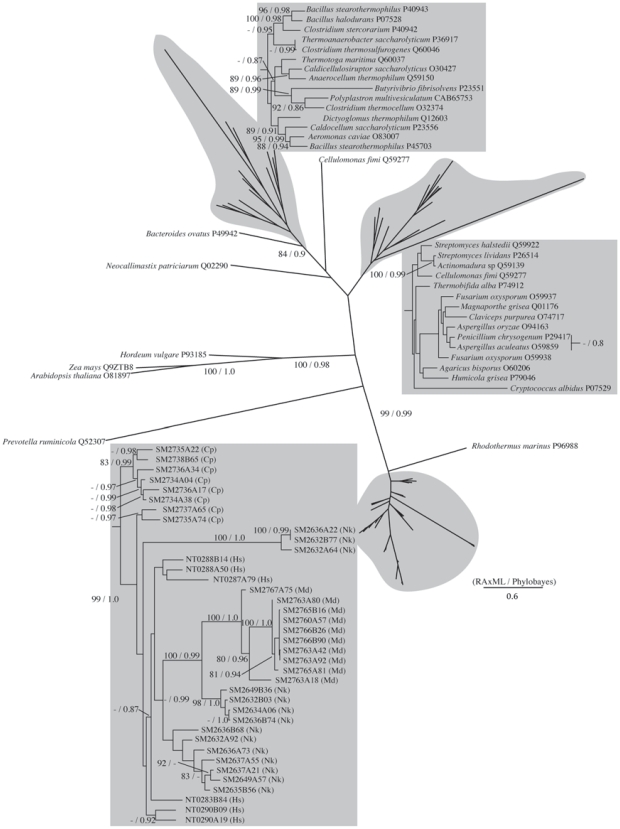
Phylogenetic tree of GHF10. Numbered clones (for example SM2735A22) are cellulase genes identified in this study from the symbiotic protists of termites. Letters in parentheses after each clone denote host termite species (Rs  =  *Reticulitermes speratus*, Nk  =  *Neotermes koshunensis*, Hs  =  *Hodotermopsis sjostedti*, Md  =  *Mastotermes darwiniensis*, Cp  =  *Cryptocercus punctulatus*). Accession numbers of reference sequences are denoted after species names.

**Figure 6:**
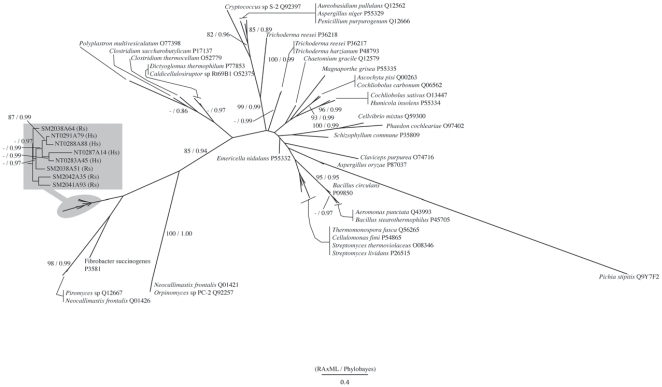
Phylogenetic tree of GHF11. Numbered clones (for example SM2038A64) are cellulase genes identified in this study from the symbiotic protists of termites. Letters in paretheses after each clone denote host termite species (Rs  =  *Reticulitermes speratus*, Nk  =  *Neotermes koshunensis*, Hs  =  *Hodotermopsis sjostedti*, Md  =  *Mastotermes darwiniensis*, Cp  =  *Cryptocercus punctulatus*). Accession numbers of reference sequences are denoted after species names.

**Figure 7:**
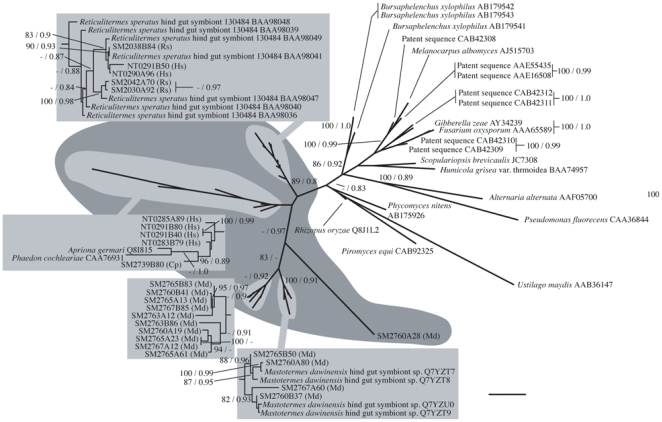
Phylogenetic tree of GHF45. Numbered clones (for example NT0285A89) are cellulase genes identified in this study from the symbiotic protists of termites. Letters in paretheses after each clone denote host termite species (Rs  =  *Reticulitermes speratus*, Nk  =  *Neotermes koshunensis*, Hs  =  *Hodotermopsis sjostedti*, Md  =  *Mastotermes darwiniensis*, Cp  =  *Cryptocercus punctulatus*). Accession numbers of reference sequences are denoted after species names.

None of the termite symbiont sequences for GHF10, 11 or 45 made monophyletic groupings that were supported in their respective analyses even though on the maximum likelihood trees each group did cluster together. The symbiont GHF10 sequences did however group with high support with a cellulase from the bacterial species *Rhodothermus marinus* (Fig. 5). The GHF11 sequences made a highly supported cluster with rumen fungal sequences and a bacterial sequence (Fig. 6) and GHF45 made a highly supported cluster with fungal (including rumen fungi) sequences (Fig. 7) with high supporting values. Although the evolutionary traits are difficult to infer, in the case of GHF10 and 11, the symbiont sequences are relatively closely related to each other in each tree. These results suggest that these genes possibly share a most recent common ancestor. In the case of GHF45, there are three well supported nodes observed in the symbiont sequence cluster. The first node contains sequences from symbionts of *Hodotermopsis* and *Reticulitermes*, the second contains *Hodotermopsis* and *Cryptocercus* symbionts and the third contains *Mastotermes* symbionts, respectively. Interestingly, the *Hodotermopsis* - *Cryptocercus* node contains the two insect cellulase genes included in this analysis [30], [31].

Our results of phylogenetic analyses with the rooted tree topologies GHF5 and 7 revealed that the lignocellulose degradation systems consist of two different evolutionary histories. The first one includes the innate functions of the protists as in GHF7. The second includes functions derived from bacteria via lateral gene transfers, such as the functions of GHF5. The former is a conventional outline of symbioses, in which a combination of distantly related organisms increases the fitness of individual organisms. The latter, a system by which the symbioses undergo dynamic processes via lateral gene transfers, can further our understanding of symbiotic relationships. Our results also indicated that the cellulolytic system was adjusted in evolutionary time by lateral gene transfers from bacteria. These aspects may open the way to a new understanding not only of symbioses, but also the interactions between diverse organisms in various evolutionary contexts.

Interestingly, both GHFs were well conserved among the lower termite lineage. This clearly reveals that these two families of enzymes work as core factors and that GHF10, 11 and 45 help these core factors for the efficient cellulose degradation of the lower termite. It also indicates that these enzyme genes are potentially useful for the construction of artificial, novel and highly efficient saccharification system without additional lignin degradation.

## Materials and Methods

### Termites and Cockroaches

*Reticulitermes speratus* was collected from the Tanzawa range (Kanagawa Japan), *Hodotermopsis sjostedti* was collected from Yaku-shima island (Kagoshima Japan), *Neotermes koshunensis* was collected from Iriomote island (Okinawa Japan), *Mastotermes darwiniensis* was collected from Darwin Australia, and *Cryptocercus punctulatus* was collected from Bear Trap Gap (North Carolina, USA). All insects were maintained in the dark with wood.

### EST Analysis

The cDNA libraries were constructed by either the biotinylated CAP trapper method [16] (*Reticulitermes speratus*) or oligo CAPPING method [32] (*Hodotermopsis sjostedti*, *Neotermes koshunensis*, *Mastotermes darwiniensis*, and *Cryptocercus punctulatus*).

Total RNA was purified from the symbiotic protists of 1,000 *R. speratus* workers using ISOGEN (Nippon Gene, Toyama, Japan) according to the manufacturer's instructions. The symbiotic protists were enriched by low-speed centrifugation (100 *g*, 3 min), lysed directly by ISOGEN reagent, and taken through the purification procedure. The total RNA obtained was further purified by Oligotex-dT super (JSR Corporation, Tokyo, Japan), a poly dT sequence conjugated latex resin, according to the method recommended by the manufacturer. mRNA was purified from the symbiotic protists of 20 *H. sjostedti* pseudergates, 50 *N. koshunensis* pseudergates, 50 *M. darwiniensis* pseudergates, and 1 specimen of *C. punctulatus*. directly by Oligotex-dT super. The cDNA libraries were constructed with purified mRNA using either method.

A method of 5′-end one-pass sequencing was performed with 910 clones (*R. speratus*), 920 clones (*H. sjostedti*), 1056 clones (*N. koshunensis*), 1021 clones (*M. darwiniensis*), and 868 clones (*C. punctulatus*), all randomly picked, using a big dye terminator sequencing kit with primer “M4” (5′-GTT TTC CCA GTC ACG AC-3′). The resulting sequences were processed manually and analyzed twice: first by FASTX against a non-redundant protein sequence database provided by the DNA Databank of Japan (version 3.2t09) without an e-value cutoff, then by blast against a merged DNA database provided by the DNA databank of Japan (release 71.0). Obtained GHF clones were sequenced by using of primer walking method. Whole length sequences were deposited into DDBJ. Accession numbers of these sequences are AB274529-AB274720. Annotation of obtained sequences was performed by fastx [18] and blast [19] analysis.

### Phylogenetic Analysis

Obtained sequences were translated to amino acid sequences and aligned with ClustalW 2.0.11 and manually inspected. Sequence alignments were analyzed using ProtTest 2.2 [33] to obtain appropriate substitution models. According to the results of ProtTest analysis, WAG+G+I+F was selected for GHF5 and 7, LG+G+F was selected for GHF10, LG+G+I+F was selected for GHF11 and WAG+G was selected for GHF45. Phylogenetic trees were inferred using the appropriate substitution model in RAxML-7.2.1 [34]. In each analysis, 4 categories of rate variation were used. To obtain supporting values, bootstrap re-sampling was performed 100 times and analyzed with the same conditions.

To obtain additional supporting values, we also performed Bayesian analyses. Phylobayes 3.1 and the CAT substitution model [35] was used for these analyses. Two independent chains were run in parallel for 10000 cycles with the pb command and default settings. The posterior distributions obtained from each run were compared with a burn-in of 1000 cycles with bpcomp command. The resulting posterior probabilities were added to each RAxML-tree.

### Statistical Test

The nested shape phylogenies were tested using the SH test [36]. The tree topology of GHF5 was manipulated manually by moving the node containing all clones to several other positions nested within sub-family 1 or sub-family 2 and keeping all subfamilies monophyletic and not breaking strongly supported nodes (sub-sub family), and then to a position basal to bacterial sub-family 1 and 2. Each resulting tree topology was combined in multi FASTA format. Each set of topologies, i.e. sub-family 1 and 2 of GHF5, was then compared using RAxML with the -f h option and the WAG+G+I+F model. Topology sets are shown in Fig. 3.
